# Exogenous leptin enhances markers of airway fibrosis in a mouse model of chronic allergic airways disease

**DOI:** 10.1186/s12931-022-02048-z

**Published:** 2022-05-24

**Authors:** Mark D. Ihrie, Victoria L. McQuade, Jack T. Womble, Akhil Hegde, Matthew S. McCravy, Cyrus Victor G. Lacuesta, Robert M. Tighe, Loretta G. Que, Julia K. L. Walker, Jennifer L. Ingram

**Affiliations:** 1 Division of Pulmonary, Allergy and Critical Care Medicine, Department of Medicine, Duke University, Durham, NC USA; 2 School of Nursing, Duke University, Durham, NC USA; 3 Durham, USA

**Keywords:** Leptin, Asthma, Airway remodeling, Sex differences

## Abstract

**Background:**

Asthma patients with comorbid obesity exhibit increased disease severity, in part, due to airway remodeling, which is also observed in mouse models of asthma and obesity. A mediator of remodeling that is increased in obesity is leptin. We hypothesized that in a mouse model of allergic airways disease, mice receiving exogenous leptin would display increased airway inflammation and fibrosis.

**Methods:**

Five-week-old male and female C57BL/6J mice were challenged with intranasal house dust mite (HDM) allergen or saline 5 days per week for 6 weeks (n = 6–9 per sex, per group). Following each HDM exposure, mice received subcutaneous recombinant human leptin or saline. At 48 h after the final HDM challenge, lung mechanics were evaluated and the mice were sacrificed. Bronchoalveolar lavage was performed and differential cell counts were determined. Lung tissue was stained with Masson’s trichrome, periodic acid-Schiff, and hematoxylin and eosin stains. Mouse lung fibroblasts were cultured, and whole lung mRNA was isolated.

**Results:**

Leptin did not affect mouse body weight, but HDM+leptin increased baseline blood glucose. In mixed-sex groups, leptin increased mouse lung fibroblast invasiveness and increased lung *Col1a1* mRNA expression. Total lung resistance and tissue damping were increased with HDM+leptin treatment, but not leptin or HDM alone. Female mice exhibited enhanced airway responsiveness to methacholine with HDM+leptin treatment, while leptin alone decreased total respiratory system resistance in male mice.

**Conclusions:**

In HDM-induced allergic airways disease, administration of exogenous leptin to mice enhanced lung resistance and increased markers of fibrosis, with differing effects between males and females.

**Supplementary Information:**

The online version contains supplementary material available at 10.1186/s12931-022-02048-z.

## Background

In the past two decades, the prevalence of asthma and obesity has increased, making them crucial public healthcare issues. In 2017, the prevalence of asthma and obesity among adults in the United States was 7.9% and 42.4%, respectively [1, 2]. Furthermore, nearly 40% of patients in the United States with asthma exhibit comorbid obesity [3]. These patients display decreased sensitivity to inhaled corticosteroid therapy and glucocorticoid resistance, which contribute to increased asthma severity, poor asthma control, and disproportionately high use of healthcare resources [4–6]. Therefore, it is critical that we understand the mechanisms underlying the relationship between asthma and obesity.

In adult asthma with comorbid obesity, two distinct endotypes may be distinguished based on age of asthma onset: early-onset asthma, which is associated with the development of asthma early in life (< 12 years of age), the treatment of which is complicated by obesity in adulthood; and late-onset asthma, which is associated with the development of asthma in adolescence or adulthood and may occur as a consequence of obesity [7]. Early-onset asthma is generally characterized by atopy, airway hyperresponsiveness, overproduction of airway mucus, type 2-cytokine-driven eosinophilic inflammation, and airway fibrosis [8]. The pathogenesis of this allergic asthma begins with immunological sensitization to environmental allergens, which derive from mold, pollen, or invertebrates such as house dust mites [9]. Briefly, antigens from these allergens are taken up by antigen-presenting cells in the lung, which activate naïve helper T cells, creating a T helper 2 (T_H_2) immune response [8]. This T_H_2 environment is characterized by the type 2 cytokines interleukins (IL)-4, -5, and -13 and antigen-specific immunoglobulin E (IgE), all of which perpetuate inflammation in the airway [8]. Eosinophils are recruited to the lung by IL-5 and eotaxin (CCL11) released by T_H_2 and mast cells, where they mediate the production of histamines and leukotrienes [8]. Chronic inflammation in asthma leads to airway remodeling and fibrosis, characterized by increased subepithelial collagen deposition [8]. Transforming growth factor (TGF)-β1 is a major mediator of fibrogenesis and promotes fibroblast proliferation and collagen synthesis [8]. Evidence from the literature indicates that the airway fibrosis component of asthma may be an important contributor to the relationship between asthma and obesity; in early-onset asthma, obesity and insulin resistance are associated with airway remodeling [10–13].

One way in which obesity may influence airway fibrosis in asthma is through the adipokine, leptin. Leptin, long known for its role in energy balance, is produced mainly by adipose tissue and acts to suppress hunger, increasing homeostatic energy consumption [14]. Circulating leptin levels are increased in patients with obesity, and these individuals may experience leptin resistance; that is, these increased leptin levels no longer promote satiety [14]. The precise mechanism through which leptin resistance occurs in these patients has yet to be determined, but may involve impaired leptin transport across the blood–brain barrier and decreased hypothalamic sensitivity to leptin [14, 15]. Mice that are leptin-deficient exhibit extreme weight gain [16]. Additionally, leptin-deficient mice exhibit impaired extracellular matrix remodeling in lung and heart, indicating a potential role for leptin in fibrotic processes [17, 18]. Furthermore, exogenous leptin administration in these mice promotes collagen production [17, 18]. Also, leptin signaling is necessary for bleomycin-induced lung fibrosis through a mechanism of peroxisome proliferator-activated receptor gamma (PPARγ) inhibition, which results in enhanced TGF-β1 signaling [19]. While these studies point to leptin’s pro-fibrotic role, the impact of increased serum leptin levels in obesity on asthma pathobiology is less well understood.

In the present study, we sought to determine how exogenous leptin administration to lean, normal chow-fed mice would impact asthmatic endpoints in chronic house dust mite (HDM)-induced allergic airway disease. We hypothesized that mice receiving exogenous leptin would display increased airway inflammation and fibrosis. Our findings indicate that increased leptin levels in obesity may contribute to airway fibrosis and hyperresponsiveness and that the impact of leptin on asthma outcomes may depend on sex.

## Methods

### Animals

Five-week-old male and female C57BL/6J mice were purchased from Jackson Laboratory (Bar Harbor, ME). Animal care and experimental protocols were approved by the Duke University Institutional Animal Care and Use Committee and carried out in accordance with the American Association for the Accreditation of Laboratory Animal Care guidelines. All mice were housed in pathogen-free facilities at Duke University. Mice were weighed and tested for glucose tolerance at weeks 0 and 6 of the protocol.

### Diet and treatments

Mice were fed normal chow (LabDiet 5053) ad libitum and received intranasal phosphate-buffered saline (PBS) or HDM allergen (Greer Laboratories (Lenoir, NC, USA), XPB70D3A2.5, lot #360924, 50 µg protein in 40 µl PBS) 5 days per week for 6 weeks under light isoflurane anesthesia. Immediately following each HDM dose, mice received subcutaneous injections of recombinant human leptin [20] (Sigma-Aldrich (St. Louis, MO) L4146) (1 mg/kg) or PBS (see Additional file 1: Fig. S1). There were 6–9 mice per sex, per treatment group (4 treatment groups), for a total of 60 mice.

### Oral glucose tolerance test

Mice were fasted for 5 h prior to the glucose tolerance test. Tails were treated with 4% lidocaine, and 200 μl of 10% glucose solution was administered by oral gavage. The mice were restrained, and blood was collected from the tail vein. Blood was collected prior to gavage and at 10-, 30-, and 90-min post-gavage. Glucose levels were determined by an Accu-Chek Performa (Roche, Basel, Switzerland) blood glucose meter.

### Lung physiology measurement

Airway responsiveness to methacholine was measured 48 h after the final HDM exposure using a computer-controlled small animal ventilator (FlexiVent, Scireq (Montreal, Canada)) as described [21]. At the conclusion of airway physiology measurements, mice were euthanized by pentobarbital overdose and tissues were immediately harvested.

### Plasma biomarkers

Immediately following airway physiology measurements and euthanasia, blood was collected by cardiac puncture and placed in a BD Microtainer lithium heparin blood tube with protease inhibitor (aprotonin and diprotin A). The blood was centrifuged at 10,000 RPM to isolate serum. Serum IgE levels were measured using Invitrogen ELISA (# 88-50460-22) (Waltham, MA) according to manufacturer protocol (plasma diluted 50×). Mouse and human leptin levels in serum were measured by DuoSet ELISA (R&D Systems, Minneapolis, MN) according to manufacturer protocol.

### Bronchoalveolar lavage (BAL)

After blood collection, lungs were lavaged with 1 ml PBS three times. BAL cells were separated by centrifugation and total cells were counted with a Scepter Handheld Automated Cell Counter (MilliporeSigma) (Burlington, MA, USA). Cells were attached to slides using a Cytospin 3 Cytocentrifuge (ThermoFisher) (Waltham, MA, USA) and fixed and stained with Easy III (Azer Scientific) (Morgantown, PA, USA). Differential cell counts were obtained by counting 200 total cells under ×200 magnification. Mouse DuoSet ELISAs for leptin, IL-5, CCL11, and TGF-β1 were obtained from R&D Systems (Minneapolis, MN, USA) and carried out according to manufacturer protocol to measure these proteins in BAL fluid.

### Lung histology

After removal of the right lung, the left lung was inflated to 25 cmH_2_O and fixed in 4% paraformaldehyde. Following fixation, the lung was embedded in paraffin, sectioned (two sections per slide), and stained with hematoxylin and eosin (H&E), Masson’s trichrome, and periodic acid-Schiff (PAS) stains.

### H&E scoring

Blinded, tiered, semi-quantitative scoring was performed on H&E-stained lung sections. Ten airways per slide were randomly selected and given a score from 0 to 4 (0 being a normal airway, and 4 being the most inflamed airway observed) for both percentage of airway circumference surrounded by inflammatory infiltrate, and depth of inflammatory infiltrate. These two scores were averaged together, and the scores from all 10 airways were averaged to derive the average inflammatory score per mouse.

### PAS scoring

Blinded, tiered, semi-quantitative scoring was performed on PAS-stained lung sections. Ten airways per slide were randomly selected and given a score from 0 to 4 for the percentage of airway circumference exhibiting positive PAS staining. The scores from the ten airways were averaged to derive the average PAS score per mouse.

### Trichrome analysis

Semi-quantitative morphometric analysis was performed on trichrome-stained lung sections. Ten circular to oval-shaped airways were randomly chosen per slide and images taken at ×200 magnification. Using ImageJ, the free-draw tool was used to trace around the circumference of the collagen layer surrounding the airway, and around the circumference of the airway lumen. These measurements give the total area and the lumen area; the lumen area is subtracted from the total area to yield the airway area. The blue trichrome stain was then isolated by using the color threshold setting to set the hue to the blue spectrum. This area of positive blue staining was measured, and the percent positive trichrome stain was derived by dividing positive stain area by the airway area and multiplying by 100.

### RNA isolation from mouse lung

At harvest, the right middle lung lobe was placed directly into 1 ml of TRI Reagent. The lobe was then homogenized using a Biospec Tissue-Tearor power homogenizer. 200 μl chloroform was added, tubes were shaken for 15 s, then incubated at room temperature for 3 min, followed by centrifugation at 12,500 RPM for 15 min at 4 °C. The upper aqueous phase was removed and placed in a new tube. 500 μl of 100% isopropanol was added to the aqueous phase, followed by 10 min incubation at room temperature, and centrifugation at 12,500 RPM for 10 min at 4 °C. Next, the supernatant was removed, leaving the RNA pellet, which was washed twice with 1 ml of 75% ethanol. Pellets were air-dried for 10 min, then were resuspended in 20 μl of RNase-free water.

### mRNA quantification

RNA concentration was measured on a NanoDrop ND-1000 (ThermoFisher) and cDNA was prepared using the Applied Biosystems High-Capacity Reverse Transcription Kit. Quantitative real-time polymerase chain reaction (qRT-PCR) was then performed using Applied Biosystems (Waltham, MA, USA) TaqMan Gene Expression Master Mix and TaqMan primers (*Gapdh* Mm99999915_g1, *Col1a1* Mm00801666_g1, *Muc5ac* Mm01276718_g1, *Muc5b* Mm00466391_m1, *Eln* Mm00514670_m1). Fold change was calculated with the delta Ct method using the PBS+saline average as the control treatment, and *Gapdh* as the endogenous control.

### Ex vivo invasion assay

Primary mouse lung fibroblast cultures were established from lung tissue digests from experimental mice. The right lower and accessory lung lobes were homogenized in Dulbecco’s Modified Eagle Medium (DMEM) containing 5% fetal bovine serum, 10 mM HEPES, 10% collagenase, and 1.4% DNAse 1 using a GentleMACS dissociator (Miltenyi Biotec) (Bergisch Gladbach, Germany). The cell suspension was filtered through a 70-μm strainer, resuspended in DMEM supplemented with 10% fetal bovine serum, and plated into 75 cm^2^ cell culture flasks for lung fibroblast cultures and grown at 5% CO_2_ and 37 °C. Fresh media was added every 2–3 days until the cells reached > 80% confluency. Fibroblasts from all groups of experimental mice were cultured and passaged under identical conditions. Invasion/migration assays were performed as described [22]. Briefly, cells at 80% confluency were resuspended in serum-free media (SFM) and seeded onto Matrigel and non-Matrigel control (BD Biosciences, San Jose, CA, USA) 24-well transwell plates (8.0 µm pore size) at 100,000 cells per insert. The cells were incubated in the presence of SFM in the apical and basal compartments of the transwell for 48 h. Following the incubation, the lower side of the inserts was stained using a Diff-Quik kit. The stained invading or migrating cells were counted under ×10 magnification.

### Statistical analysis

Statistical analyses were performed in GraphPad Prism 9. The distribution of the data was examined visually, outliers were tested with the Robust Regression and Outlier Removal method [23] and were removed where appropriate. Data were evaluated for normality via the Anderson–Darling test. Parametric or non-parametric tests were employed accordingly (one, two, or three-way ANOVA or Kruskal–Wallis), with appropriate post-test (specific tests are noted in figure legends). Significance is denoted by p < 0.05, and all data are presented as the mean ± SEM.

## Results

**Fig. 1:**
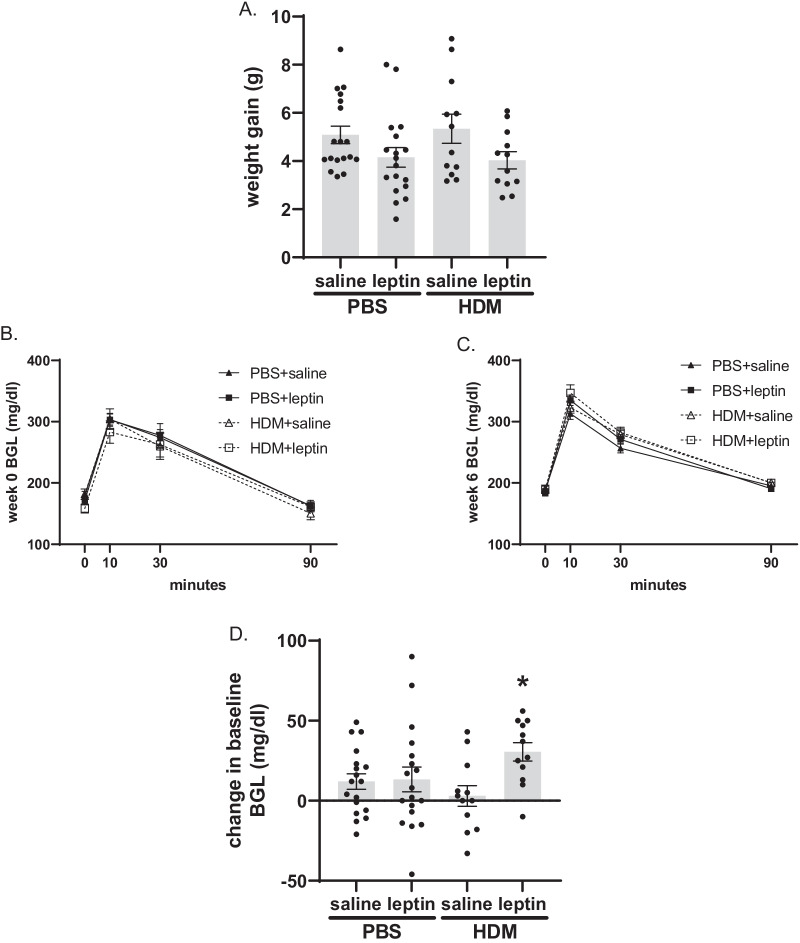
Weight gain and glucose tolerance. **A** Change in mouse weight from week 0 to week 6, n = 12–18 mice per group. **B**, **C** Glucose tolerance curves at **B** week 0 and **C** week 6. **D** Change in baseline blood glucose level from week 0 to week 6, n = 12–18 mice per group. *p < 0.05 HDM+saline vs. HDM+leptin by two-way ANOVA with Tukey’s post hoc test

Exogenous leptin administration in HDM exposed mice increases baseline blood glucose levels but does not affect weight gain. Overall, serum leptin levels did not significantly differ between exposure conditions (see Additional file 1: Fig. S2A); yet baseline levels of circulating leptin in control mice (intranasal PBS with subcutaneous saline) were significantly elevated in male mice compared to female mice (see Additional file 1: Fig. S2B). Mice were weighed weekly throughout the course of the 6-week exposure to leptin and HDM, and no significant changes in cumulative weight gain were seen between groups. However, the leptin-treated groups tended to have reduced weight gain compared to saline-treated groups (Fig. 1A). Glucose tolerance was assessed at week 0 prior to beginning treatment and at week 6. While no differences in glucose response were seen between conditions at week 0 or week 6 (Fig. 1B, C), the change in baseline blood glucose levels over the course of the protocol was significantly higher in HDM+leptin exposed mice compared with PBS+saline (Fig. 1D). Area under the curve analysis was performed on glucose tolerance data, however, it indicated no significant changes between conditions (see Additional file 1: Fig. S3).

**Fig. 2:**
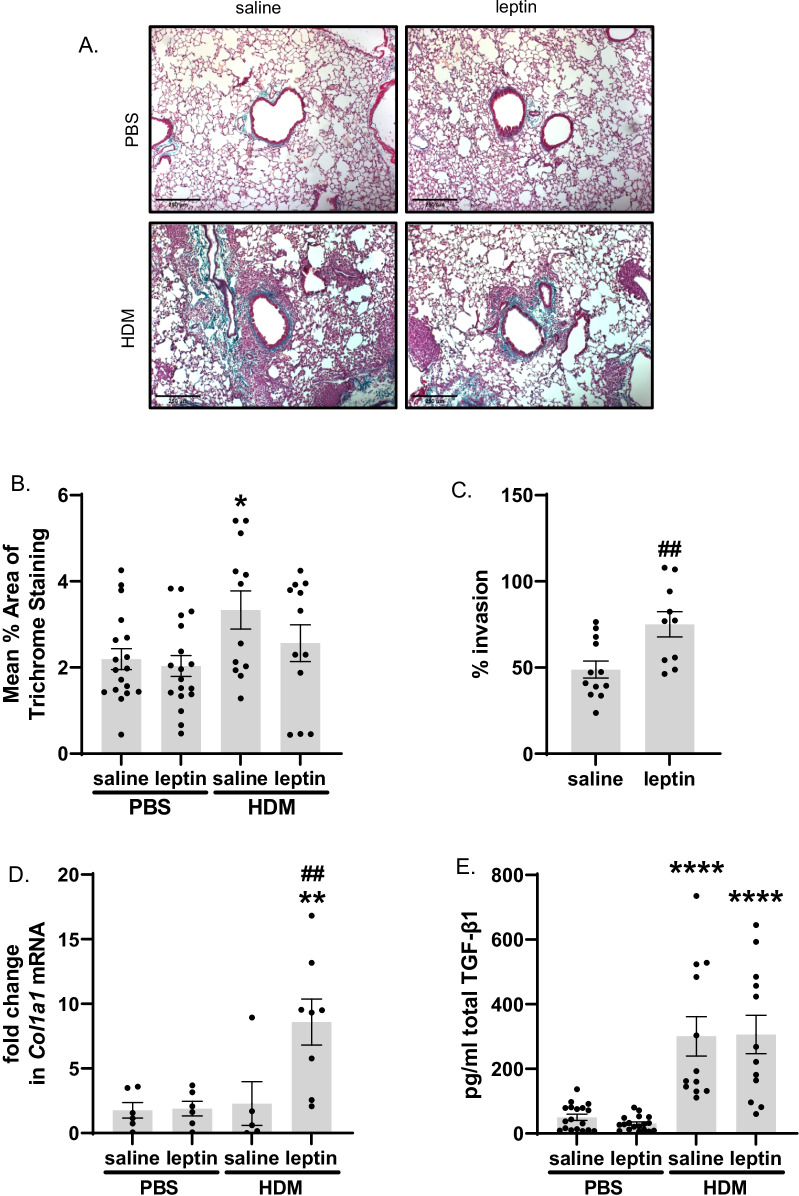
Changes in profibrotic endpoints. **A** Representative images of Masson’s trichrome-stained mouse lung sections taken at ×100 magnification, scale bar = 250 μm. Blue staining around the airways indicates positive trichrome staining. **B** Quantification of mean Masson’s trichrome staining, n = 12–18 mice per group, 10 airways per mouse. **C** Mean percent invasion in control treated fibroblasts (number of invading cells divided by number of migrating cells × 100) lung fibroblasts determined by Matrigel invasion assay, n = 10–12 mice per group, cultures performed in duplicate. **D** *Col1a1* mRNA expression in whole lung, n = 5–8 mice per group. **E** Total TGF-β1 protein levels in BAL n = 12–18 mice per group; *p < 0.05, **p < 0.01, ****p < 0.0001 vs. corresponding PBS treatment, ^##^p < 0.01 vs. saline by two-way ANOVA with Šidák correction

Markers of airway fibrosis are increased by leptin treatment. Representative photomicrographs of Masson’s trichrome-stained lung sections indicate typically observed increased airway inflammation and peribronchiolar collagen deposition in HDM-exposed groups compared to PBS-exposed groups (Fig. 2A). Quantification of peribronchiolar trichrome staining revealed a significant increase in collagen deposition with HDM exposure compared with PBS control (Fig. 2B). Lung fibroblast invasiveness was evaluated ex vivo via invasion/migration assay, which showed that leptin treatment significantly increased baseline percent invasion of mouse lung fibroblasts relative to saline treatment (Fig. 2C). There were no effects of HDM exposure on fibroblast invasiveness (Additional file 1: Fig. S4D) Additionally, whole lung type I collagen (*Col1a1*) mRNA levels displayed a similar response, with leptin significantly increasing levels over saline in lung fibroblasts isolated from HDM-exposed mice (Fig. 2D). Levels of elastin (*Eln*) mRNA were also measured but no significant differences between conditions were observed (see Additional file 1: Fig. S4C). Total TGF-β1 protein levels in BAL fluid indicated a significant HDM effect, however, no change was seen with leptin treatment (Fig. 2E).

**Fig. 3:**
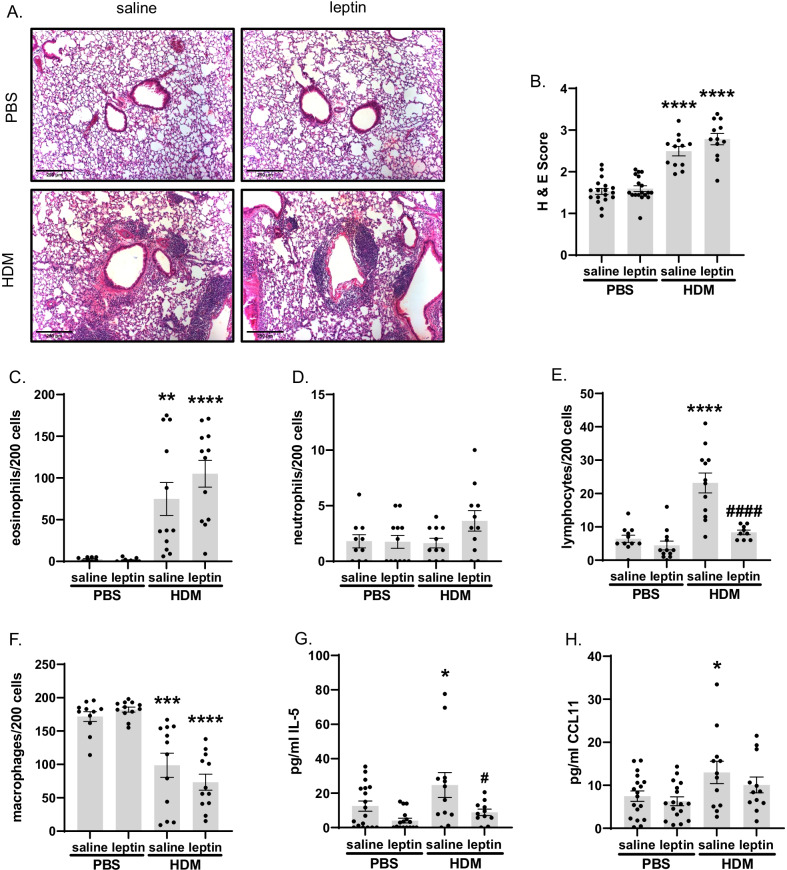
Changes in peribronchial inflammation and BAL cellularity. **A** Representative images of H&E-stained mouse lung sections taken at ×100 magnification, scale bar = 250 μm. **B** Mean peribronchial inflammation score of H&E-stained lung sections, n = 12–18 mice per group, 9 airways per mouse. **C**–**F** Relative numbers of **C** eosinophils, **D** neutrophils, **E** lymphocytes, and **F** macrophages in BAL were evaluated, n = 8–12 mice per group. **G** IL-5 and **H** CCL11 levels measured in BAL fluid, n = 12–18 mice per group. *p < 0.05, **p < 0.01, ***p < 0.001, ****p < 0.0001 vs. corresponding PBS treatment; ^#^p < 0.05, ^####^p < 0.0001 vs. HDM+saline by two-way ANOVA with Šidák correction

HDM increases airway inflammation and BAL eosinophil and lymphocyte numbers, while leptin decreases BAL lymphocytes. Scoring of peribronchiolar inflammation revealed that HDM-challenged mice exhibited significantly enhanced airway inflammation independent of leptin treatment (Fig. 3B). Total counts of BAL fluid eosinophils were significantly increased in HDM-challenged mice compared to saline-treated mice, independent of leptin treatment (Fig. 3C). In contrast, leptin treatment significantly reduced HDM-induced BAL lymphocyte numbers compared to HDM/saline-treated mice (Fig. 3E). Changes in eosinophil and lymphocyte numbers were reflected as decreases in macrophage numbers, but numbers of airway neutrophils were not significantly different between conditions (Fig. 3D, F). HDM challenge induced significantly increased IL-5 and CCL11 levels in BAL fluid compared to saline-treated mice; further, leptin treatment significantly reduced IL-5, but not CCL11, in HDM-exposed mice compared to HDM alone (Fig. 3G, H).

**Fig. 4:**
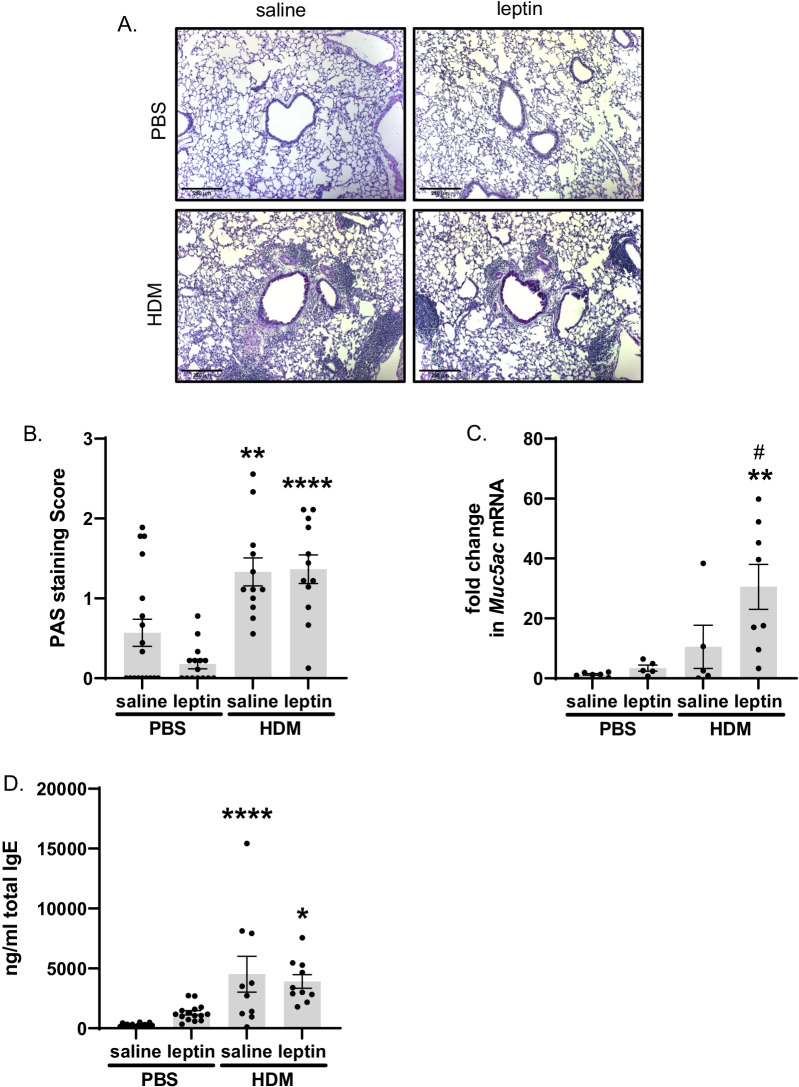
Changes in airway mucus production and serum IgE. **A** Representative images of PAS-stained mouse lung sections taken at ×100 magnification, scale bar = 250 μm. Dark purple staining in the airway epithelium indicates positive PAS staining. **B** Mean peribronchial mucus score of PAS-stained lung sections, n = 12–18 mice per group, 9 airways per mouse. **C** Changes in whole lung *Muc5ac* mRNA expression, n = 5–8 mice per group. **D** Changes is total serum IgE levels as measured by ELISA, n = 10–16 mice per group. *p < 0.05, **p < 0.01, ****p < 0.0001 vs. corresponding PBS treatment by two-way ANOVA with Šidák correction

HDM exposure with exogenous leptin administration increases airway mucus production, *Muc5ac* mRNA expression, and serum IgE. Scoring of peribronchiolar mucin in PAS-stained lung sections demonstrated that HDM exposure significantly enhanced airway mucus production alone and with leptin administration, while leptin alone had no effect. (Fig. 4B). *Muc5ac* mRNA levels in whole lung were significantly increased in the HDM+leptin group, but not HDM alone (Fig. 4C), while no significant differences between groups were seen in MUC5AC protein levels or *Muc5b* mRNA expression (see Additional file 1: Fig. S4A, B). Serum IgE levels were significantly increased by HDM in both saline and leptin-administered mice; while leptin alone did not significantly affect IgE levels, there was an upward trend with leptin administration (Fig. 4D).

**Fig. 5:**
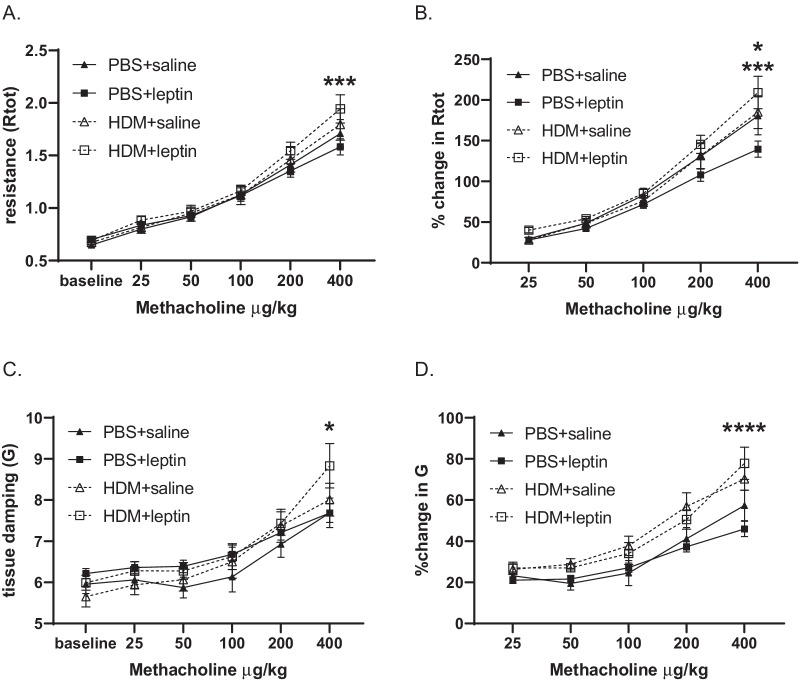
Changes in lung mechanics. **A**, **B** Change in total lung resistance in response to methacholine, both **A** absolute and **B** percent change, n = 11–16 mice per group. ***p < 0.001 PBS+leptin vs. HDM+leptin and *p < 0.05 PBS+saline vs. PBS+leptin by two-way ANOVA with Tukey’s post hoc test. **C**, **D** Change in tissue damping in response to methacholine, both **C** absolute and **D** percent change, n = 11–16 mice per group. *p < 0.05 PBS+leptin vs. HDM+leptin and ****p < 0.0001 PBS+leptin vs. HDM+leptin by two-way ANOVA with Tukey’s post hoc test

Total lung resistance and tissue damping are increased by HDM exposure with exogenous leptin administration. Lung mechanics were assessed at 6 weeks of chronic HDM exposure and leptin administration. In mixed-sex groups, total respiratory resistance (Rtot) was significantly increased in the HDM+leptin group compared with the leptin alone group, but not compared with the HDM alone group (Fig. 5A). Percent change in total respiratory resistance was also significantly increased by HDM+leptin compared with leptin alone, however, this parameter was also significantly decreased by treatment with leptin alone compared with saline control (Fig. 5B). Similarly, tissue damping (G) was significantly increased by HDM+leptin compared with leptin alone (Fig. 5C, D). Newtonian resistance (Rn), elastance (E), and tissue elastance (H) were also measured, however, no significant changes between groups were seen (see Additional file 1: Fig. S5).

**Fig. 6:**
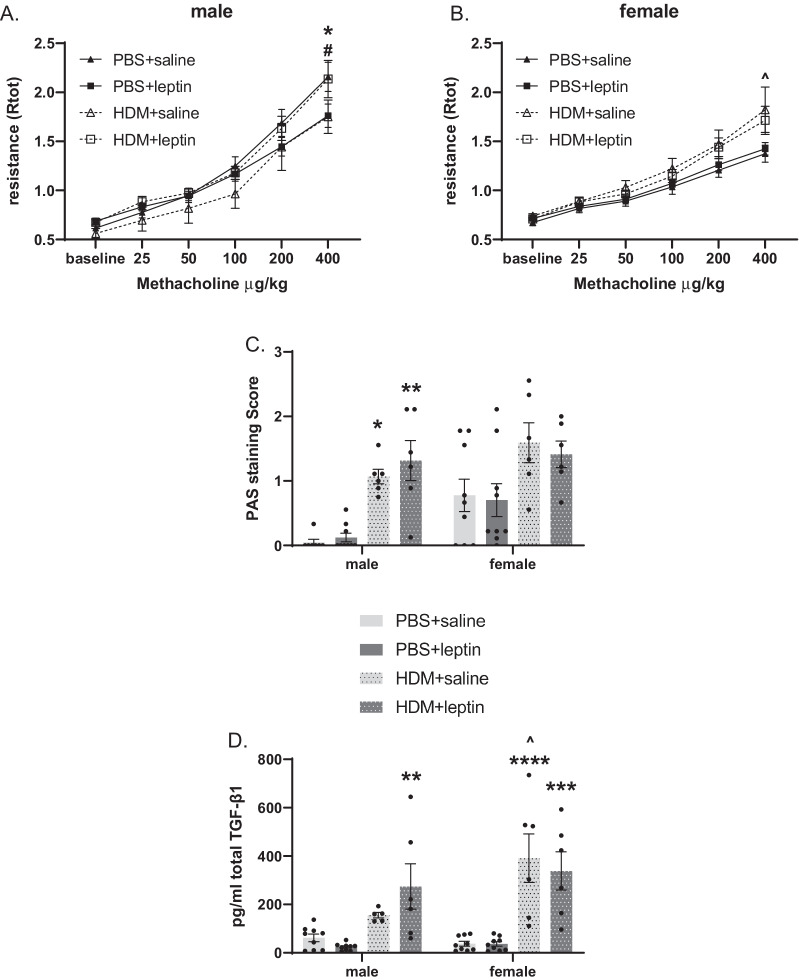
Sex differences. **A**, **B** Comparison of changes in total lung resistance between treatments in **A** males and **B** females, n = 3–8 mice per group. *p < 0.05 PBS+saline vs. PBS+leptin, ^#^p < 0.05 PBS+leptin vs. HDM+leptin, ^^^p < 0.05 PBS+saline vs. HDM+saline by two-way ANOVA with Tukey’s post hoc test. **C** Sex differences in airway mucus were evaluated by scoring of peribronchial mucus in PAS-stained lungs, n = 6–9 mice per group, 9 airways per mouse. **D** Sex differences in TGB-β1 levels in BAL evaluated by ELISA, n = 5–9 mice per group. *p < 0.05 **p < 0.01, ***p < 0.001, ****p < 0.0001 vs. corresponding PBS treatment by three-way ANOVA with Šidák correction, ^^^p < 0.05 vs. male of the same treatment by three-way ANOVA with Šidák correction

Sex-dependent differences are present in HDM-induced airway hyperresponsiveness, mucus production, and TGF-β1 levels with evidence of leptin-specific effects. While HDM exposure alone did not increase Rtot in male mice, it significantly increased Rtot in females, independent of leptin treatment (Fig. 6B). Furthermore, leptin treatment had no effects on Rtot in female mice (Fig. 6B). Conversely, leptin treatment in male mice significantly decreased total respiratory resistance (Rtot) in the absence of HDM, but when combined with HDM exposure an increase in Rtot was seen (Fig. 6A). While HDM challenge resulted in significantly increased peribronchiolar PAS staining regardless of leptin treatment in mixed-sex groups (Fig. 4A), only male mice, not female, exhibited significantly increased PAS staining with HDM exposure. (Fig. 6C). Conversely, while both male and female mice showed increased total TGF-β1 protein levels in BAL with HDM+leptin, female mice, but not male, had significantly higher TGF-β1 levels in response to HDM alone (Fig. 6D).

## Discussion

In this study, we investigated the effects of leptin in a mouse model of chronic, allergic airways disease. While several studies have previously investigated the role of leptin in lung injury and disease, this study is the first to examine the effects of leptin on chronic, HDM-induced allergic airway disease. Importantly, this study demonstrates the impact of leptin on airway physiology, fibrosis, and inflammation, in addition to examining sex-dependent differences. The present study’s key findings were that while chronic HDM exposure causes airway inflammation, in mixed-sex groups, significant increases in airway resistance only occurred with the addition of exogenous leptin, and leptin increased markers of airway fibrosis and remodeling. Therefore, this study provides new insight into how leptin may directly affect lung inflammation and fibrosis in asthma.

A recent study by Kurokawa et al. examined the effect of exogenous leptin administration on IL-33-induced lung inflammation in both wildtype and leptin deficient mice [24]. While this study differs from the present study in several ways (it utilizes a different, more short term asthma model, only female mice, and murine exogenous leptin), they also measured airway physiology using the FlexiVent system and found that in wildtype mice, exogenous leptin, when combined with IL-33, increased Rrs (equivalent to Rtot), and did not affect Ers, Rn, and H, which is consistent with our findings [24]. G was not affected by exogenous leptin in their study, while in the present study G was increased by HDM+leptin. While increases in Rtot alone indicate increased central and peripheral airway resistance due to airway narrowing and/or closure, the increase in tissue damping (G) seen with HDM+leptin treatment, along with the lack of change in tissue elastance (H), indicates that central airway narrowing, rather than airway closure, may be resulting from leptin treatment. Furthermore, Kurokawa et al. found that serum leptin levels return to baseline 24 h after injection, which explains why we did not see elevated leptin levels in the present study [24]. Another notable similarity between these studies is that exogenous leptin administration did not alter BAL eosinophil levels induced by the respective asthma models, but exogenous leptin did significantly decrease BAL IL-5 levels [24].

Several additional studies have investigated the role of leptin in lung disease and injury by utilizing exogenous leptin administration. Shore et al. found that leptin did not affect BAL eosinophil numbers, which is consistent with our present study [25]. Conversely, Shore et al. saw no changes in BAL IL-5 levels with leptin treatment, while we found that leptin significantly decreased HDM-induced IL-5 in BAL [25]. Leptin has been shown to play a role in mediating inflammation after cigarette smoke exposure; leptin-deficient mice exhibit increased neutrophil numbers and decreased CD4^+^ and CD8^+^ cells compared with wild-type mice, while exogenous leptin administration restores inflammatory cell profiles [26]. In the chronic asthma model utilized in the present study, leptin did not alter BAL neutrophils, however, leptin did significantly decrease HDM-induced BAL lymphocyte numbers, suggesting an immunomodulatory role. Interestingly, leptin administered intranasally has been shown to attenuate LPS-induced lung injury [27, 28]. Furthermore, leptin can restore impaired host defense following fasting [29]. These studies indicate leptin can augment immune responses, which may, in the context of asthma, be detrimental. However, in our chronic allergen-challenge study, leptin did not affect lung inflammation but rather had specific effects on lung resistance and remodeling endpoints. A study by Jain et al. found that leptin enhances transcription of profibrotic genes downstream of TGF-β1 by human lung fibroblasts in vitro [19]. We similarly saw an increase in *Col1a1* expression in mice treated with HDM and leptin, as well as increased lung fibroblast invasion in vitro, indicating a profibrotic fibroblast phenotype. Additionally, though we did not see a leptin effect on PAS staining for airway mucus, we did observe that leptin increased *Muc5ac* mRNA; previous studies show that leptin can increase IL-13-induced MUC5AC production in human bronchial epithelial cells [30]. Finally, as leptin plays a key role in energy homeostasis, it may seem surprising that we did not see changes in mouse weight with exogenous leptin administration; however, it has been reported previously that leptin administration to mice does not significantly alter body weight [20, 24].

Sex differences in human asthma are well known; women have increased asthma prevalence and are more likely to have severe asthma [31]. Additionally, mouse models show that estrogen increases airway inflammation; however, some studies show that female mice have enhanced inflammation, while others show no change [31]. It is also known that circulating leptin levels are higher in females, and that leptin levels generally correlate with body fat percentage [32, 33]. Evidence also shows that sex hormones may affect the metabolic actions of leptin, with estrogens enhancing the ability of leptin to inhibit eating [34]. Since little is known about how leptin influences asthma, it is also unclear how sex may affect leptin-induced changes. Surprisingly, in our study, despite the combination of HDM+leptin causing increased Rtot in mixed-sex groups, leptin alone significantly decreased Rtot in male mice, while female mice showed no effects from leptin treatment and had a greater response to HDM. One limitation of our study regarding sex-dependent differences is that we did not track the estrus cycle of female mice, so it is possible that this influenced the responses we saw in females. In general, we saw that male mice had enhanced responsiveness to methacholine challenge in both control and HDM-exposed groups compared with female mice. Sex differences in airway responsiveness are variable and dependent on mouse strains. Still, previous literature shows that naïve male C57BL/6 mice have enhanced responsiveness compared to females, which is consistent with our study [35, 36]. Furthermore, we saw that only male mice had significantly increased airway mucus production. The lack of significance in female mice may be due to the relatively higher baseline levels of mucus observed in these mice compared with males. Lastly, female mice had significantly higher levels than males, while only male mice exhibited a significant leptin effect. This, taken together with the changes in AHR seen in male mice, suggests that leptin may play a greater role in modulating asthma outcomes in males compared to females.

One limitation of this study is the use of subcutaneous leptin injections instead of direct administration of leptin to the lung through a method such as oropharyngeal aspiration, which might allow for a better understanding of leptin’s direct effects on the lung. It was recently shown that leptin administered intranasally alleviates sleep-disordered breathing, while intraperitoneal leptin injection had no effect [37, 38]. This highlights the importance of the leptin exposure route. Additionally, it is possible that leptin administered subcutaneously could have effects on the central nervous system (CNS), which could then, in turn, impact asthma outcomes. A recent study by Do et al. reports that leptin can act on the CNS to mediate breathing mechanics [39]. Therefore, while subcutaneous leptin administration may mimic increased systemic leptin levels present in obesity, it may be insufficient to elucidate the direct effects of leptin on the lungs. Thus, transgenic overexpression of leptin targeted to the mouse lung may be a more effective way to evaluate leptin’s direct effects on the lung.

## Conclusion

Our work demonstrates that administration of exogenous leptin to mice can enhance markers of fibrosis and increase lung resistance in a model of allergic airways disease. Furthermore, this study revealed sex differences in response to leptin, indicating that increased leptin levels in obesity may affect males more than females. The lack of significant changes in HDM-induced lung inflammation, together with increased markers of fibrosis, indicates that lung fibrosis may contribute to the observed leptin-induced changes in lung resistance. Further work will be needed to fully elucidate the mechanisms through which leptin affects asthma and fibrosis.

## Supplementary Information

**Additional file 1: Figure S1.** Schematic of mouse dosing protocol. **Figure S2.** Additional ELISA data. Mouse leptin levels measured in serum, presented as **A** mixed-sex groupings and **B** by-sex groupings n = 12–17 mice per group for mixed-sex groups, n = 6–9 mice per group per sex. ^^^^p < 0.01 vs. male by three-way ANOVA with Šidák correction. **Figure S3.** Change from week 0 to week 6 of the area under the glucose tolerance curves. No significant differences were observed between treatment groups. **Figure S4.** Additional qPCR and ELISA data from whole lung; and fibroblast invasion. MUC5AC was significantly increased (*p < 0.05) by HDM exposure. No significant changes in were seen in *Eln* and *Muc5b* mRNA expression. **Figure S5.** Additional lung physiology measurements in mixed-sex groups: **A** Newtonian resistance, **B** tissue elastance, and **C** elastance. No significant changes were observed, n = 11–16 mice per group.

## Data Availability

The datasets used and/or analysed during the current study are available from the corresponding author on reasonable request.

## Abbreviations

HDM: House dust mite
BAL: Bronchoalveolar lavage
Th2: T helper type 2
IL: Interleukin
IgE: Immunoglobulin E
TGF: Transforming growth factor
PPAR: Peroxisome proliferator activated receptor
PBS: Phosphate buffered saline
H&E: Hematoxylin and eosin
PAS: Periodic acid-Schiff
qRT-PCR: Quantitative reverse transcriptase polymerase chain reaction
DMEM: Dulbecco’s Modified Eagle Medium
SFM: Serum-free media
Rtot: Total lung resistance
G: Tissue damping
Rn: Newtonian resistance
E: Elastance
H: Tissue elastance
CNS: Central nervous system
