# Supplementary material for: Exogenous leptin enhances markers of airway fibrosis in a mouse model of chronic allergic airways disease
Source: Respir Res. 2022 May 24;23:131. doi: 10.1186/s12931-022-02048-z (PMC9131622; doi:10.1186/s12931-022-02048-z)
Supplement: Supplementary file 1 — Additional file 1: Figure S1. Schematic of mouse dosing protocol. Figure S2. Additional ELISA data. Mouse leptin levels measured in serum, presented as A mixed-sex groupings and B by-sex groupings n = 12–17 mice per group for mixed-sex groups, n = 6–9 mice per group per sex. ^^p < 0.01 vs. male by three-way ANOVA with Šidák correction. Figure S3. Change from week 0 to week 6 of the area under the glucose tolerance curves. No significant differences were observed between treatment groups. Figure S4. Additional qPCR and ELISA data from whole lung; and fibroblast invasion. MUC5AC was significantly increased (*p < 0.05) by HDM exposure. No significant changes in were seen in Eln and Muc5b mRNA expression. Figure S5. Additional lung physiology measurements in mixed-sex groups: A Newtonian resistance, B tissue elastance, and C elastance. No significant changes were observed, n = 11–16 mice per group. [file 12931_2022_2048_MOESM1_ESM.pptx]

## Slide 1
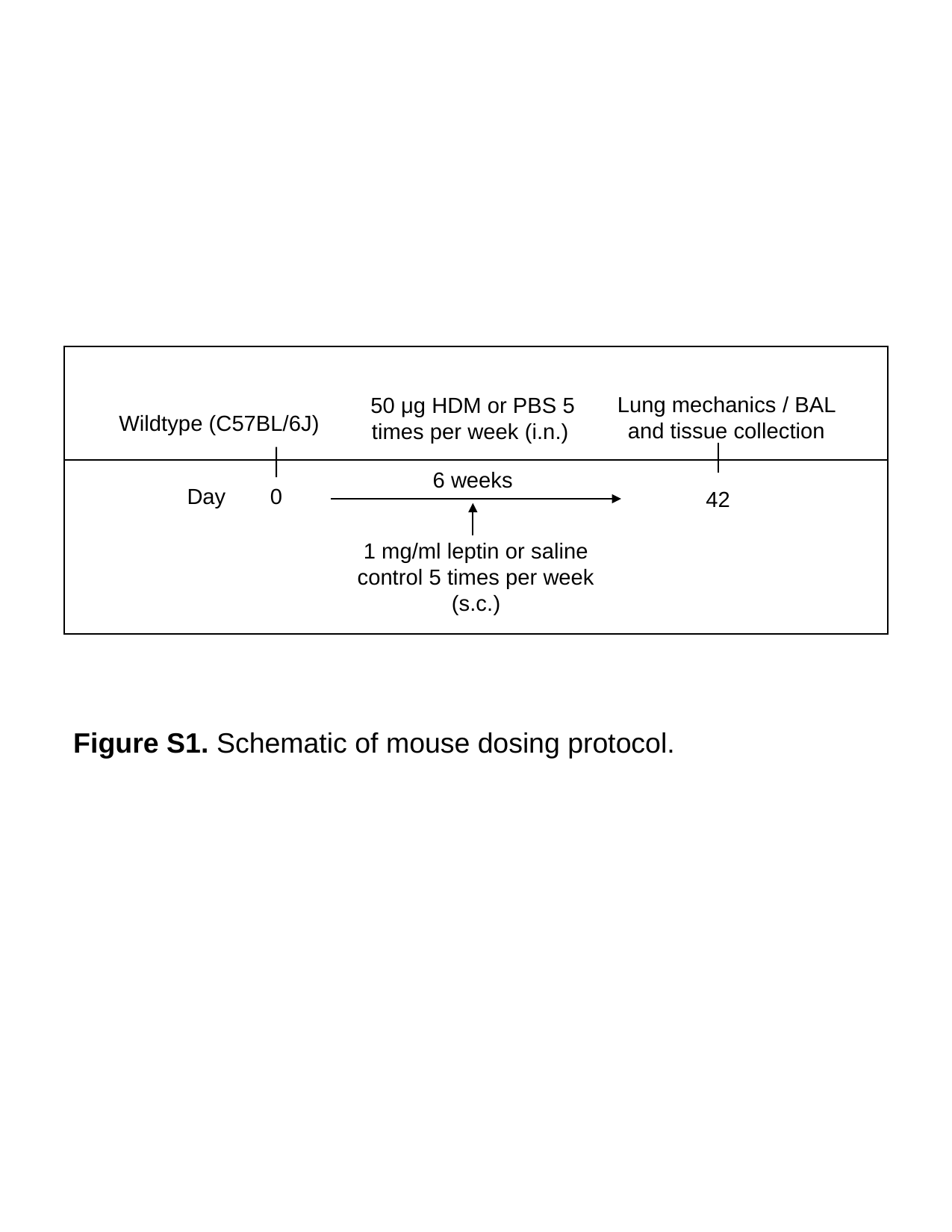

Lung mechanics / BAL and tissue collection
50 μg HDM or PBS 5 times per week (i.n.)
Wildtype (C57BL/6J)
6 weeks
Day
0
42
1 mg/ml leptin or saline control 5 times per week (s.c.)
Figure S1. Schematic of mouse dosing protocol.

## Slide 2
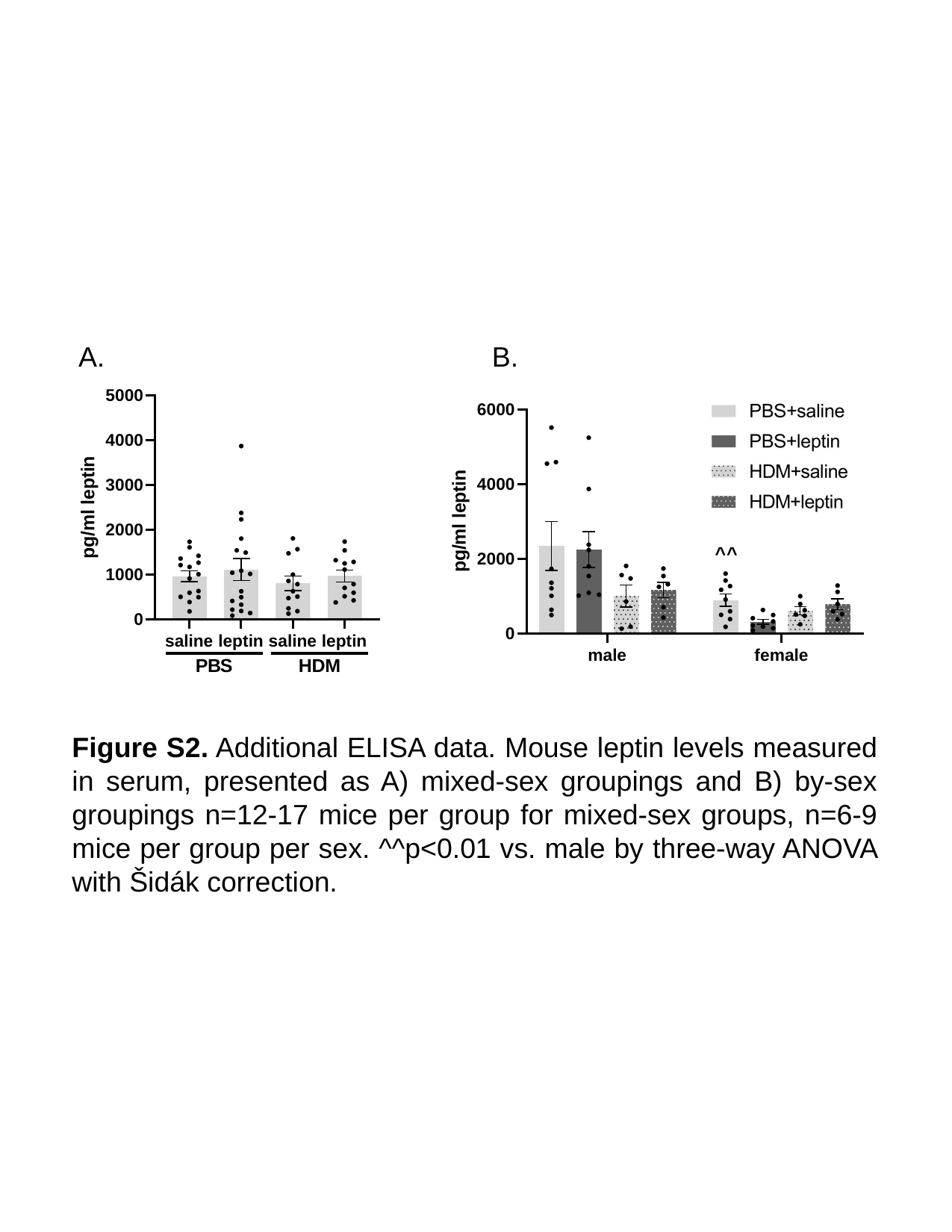

A.
B.
Figure S2. Additional ELISA data. Mouse leptin levels measured in serum, presented as A) mixed-sex groupings and B) by-sex groupings n=12-17 mice per group for mixed-sex groups, n=6-9 mice per group per sex. ^^p<0.01 vs. male by three-way ANOVA with Šidák correction.

## Slide 3
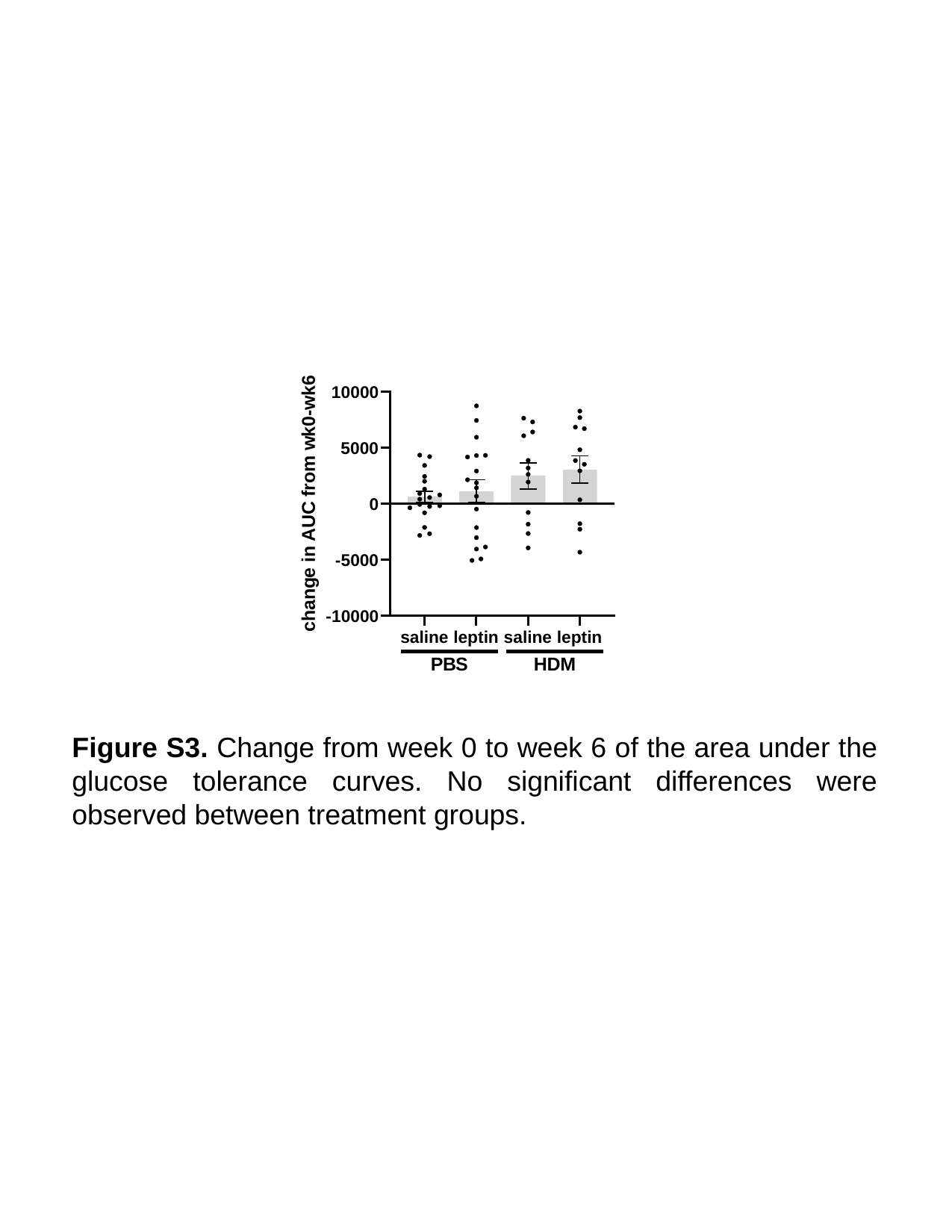

Figure S3. Change from week 0 to week 6 of the area under the glucose tolerance curves. No significant differences were observed between treatment groups.

## Slide 4
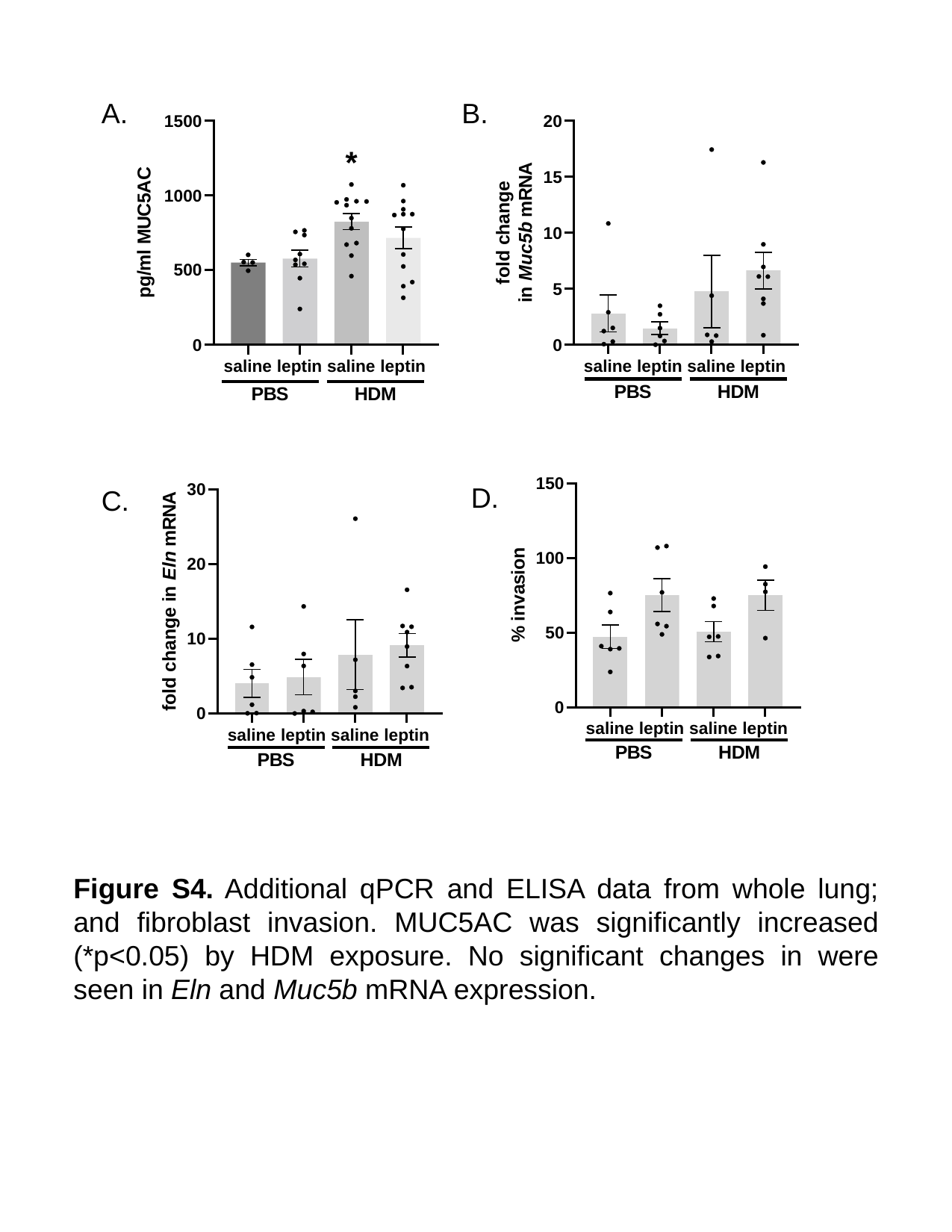

A.
B.
D.
C.
Figure S4. Additional qPCR and ELISA data from whole lung; and fibroblast invasion. MUC5AC was significantly increased (*p<0.05) by HDM exposure. No significant changes in were seen in Eln and Muc5b mRNA expression.

## Slide 5
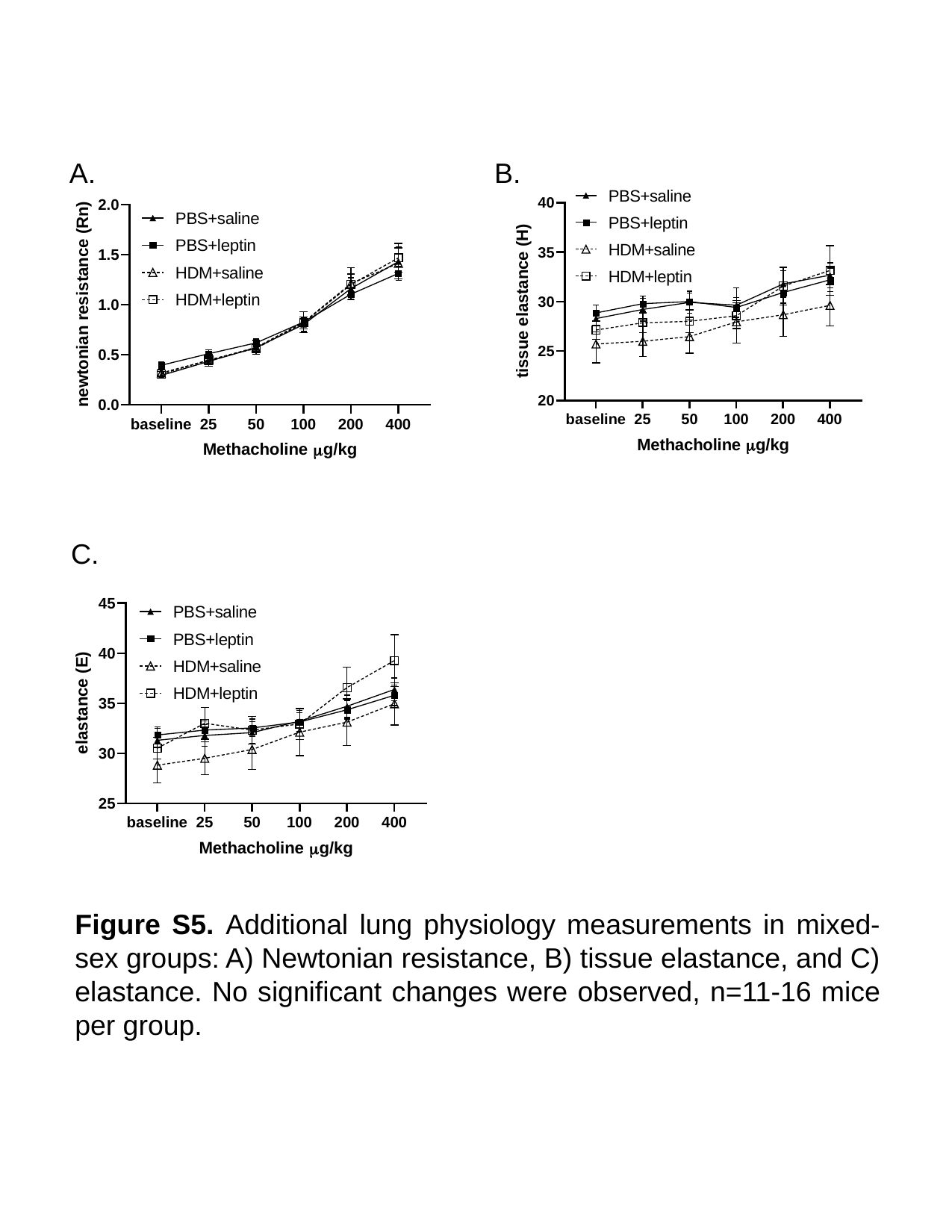

A.
B.
C.
Figure S5. Additional lung physiology measurements in mixed-sex groups: A) Newtonian resistance, B) tissue elastance, and C) elastance. No significant changes were observed, n=11-16 mice per group.
